# COVID-19 Emergency and Post-Emergency in Italian Cancer Patients: How Can Patients Be Assisted?

**DOI:** 10.3389/fonc.2020.01571

**Published:** 2020-08-11

**Authors:** Anna Crispo, Concetta Montagnese, Francesco Perri, Maria Grimaldi, Sabrina Bimonte, Livia Silvia Augustin, Alfonso Amore, Egidio Celentano, Marilena Di Napoli, Marco Cascella, Sandro Pignata

**Affiliations:** ^1^Epidemiology and Biostatistics Unit, Istituto Nazionale Tumori, IRCCS Fondazione G. Pascale, Naples, Italy; ^2^Head and Neck Medical and Experimental Oncology Unit, Istituto Nazionale Tumori, IRCCS Fondazione G. Pascale, Naples, Italy; ^3^Department of Anesthesia and Pain Medicine, Istituto Nazionale Tumori, IRCCS Fondazione G. Pascale, Naples, Italy; ^4^Medical Oncology Unit, Istituto Nazionale Tumori, IRCCS Fondazione G. Pascale, Naples, Italy

**Keywords:** COVID-19, coronavirus, cancer patients, telemedicine, infections

## Abstract

Italy and worldwide are experiencing an outbreak of a new coronavirus-related disease, named COVID-19, declared by the WHO COVID-19 a pandemic. The fragility of cancer patients is well-known, with many cases affecting aged patients or those with several comorbidities that frequently result in a loss of independency and functionality. Therefore, cancer patients have been greatly affected by this health emergency and, due to their vulnerability to COVID-19, oncologic patient visits have been often delayed or canceled leading to possible under-treatment. Different solutions can be adopted for reducing travels to cancer screening centers and the overall impact of cancer screening visits. As a consequence, it has been recommended that, when possible, the follow-up visits for cancer patients treated with oral anticancer drugs could be performed telematically. Furthermore, many patients refuse hospital visits, even if necessary, because of fear of contagion. Moreover, in some regions in Italy even the very first non-urgent visits have been postponed with the consequent delay in diagnosis, which may negatively affect disease prognosis. For these reasons, new approaches are needed such as the telemedicine tool. Throughout organized and appropriate tools, it would be possible to manage patients’ visits and treatments, to avoid the dangerous extension of waiting lists when the standard activities will resume. In this context, a number of hospital visits can be substituted with visits at small local health centers, and general practitioners’office, taking in turn, advantage of well-defined telemedicine path which will be developed in the post-emergency phase.

## Background

### Epidemiology

Italy and worldwide are experiencing an outbreak of a new coronavirus-related disease named COVID-19 due to SARS-CoV2 virus. Cases that develop severe pneumonitis are characterized by dyspnea, cyanosis and fever, which may configure the clinical framework of Acute Distress Respiratory Syndrome (ARDS) (1, 2). Because COVID-19 spread to more than 100 countries and accounted for several tens of thousands of cases within a few months, WHO declared COVID-19 a pandemic (3).

Italy currently is the second European country after Spain for confirmed COVID-19 cases (197,675 and 207,634, respectively), and the second country in the world for COVID-19 death after United States (26,644 and 47,980, respectively) (4). Of note, although in Italy the outbreak of SARS-CoV-2 infection grew progressively from end of Feb 2020 to end of Mar 2020 over the entire national territory, large regional differences were recorded.

On March 9, 2020, the lockdown was extended for the entire national territory and progressively severe limitations were adopted. The dynamics of the epidemics followed a geographical differentiation, with a North to South gradient that is likely to depend on the different timing of onset of the local outbreaks before the implementation of national containment measures.

A report from the Italian Superior Institute of Health based on 3,200 patients who died of SARS-CoV-2 related mortality to old age and comorbidities including cancer (5). Patients suffering from oncologic or onco-hematologic pathologies, as well as other diseases associated with immunosuppression (e.g., congenital immunodeficiency, solid organ transplants or hematopoietic stem cells, autoimmune diseases following immunosuppressive treatment), are particularly at risk for morbidity and mortality from respiratory viral infections such as influenza for which the risk of hospitalization of cancer patients is approximately four times higher than in healthy subjects of comparable age (6). Although the data are currently extremely limited, it seems that patients with oncologic or onco-hematologic diseases are exposed either to greater risk of contracting SARS-CoV-2 infection or to have a worse prognosis. In fact, these patients are characterized by a greater risk of events such as hospitalization in intensive care unit and/or exitus.

As of now no validated vaccines or antiviral drugs against SARS-CoV-2 infection exist (7), the hospital in our country have provided and establish dedicated areas (e.g., waiting rooms) for cancer patients. Preventive measures have been doubled by implementing active-surveillance that allows cases to be identified earlier, isolating them according to appropriate management and containment procedures. Another measure to reduce contagion of vulnerable patients was to postpone visits for non-urgent cancer patients. This however, poses the question on how to best follow patients in their homes (8, 9). Finally in order to change the clinical practice for oncologic patients in terms of assistance and controls, a multidisciplinary team is needed to improve the diagnostic and therapeutic management for cancer patients (10).

### Telemedicine

The term “telemedicine” was created with the aim of identifying a particular sector of application of Information and Communication Systems, ICT, with the aim of providing a better performance for healthcare activities. The term is used both to describe computer and telecommunication systems that allow people to work together over time and space, and to refer generally to the use of information technology in medicine. Worldwide, there has been an important increase in the use of telemedicine in the health sector in recent years. Telecommunication systems provide remote healthcare by improving access to care and changing its organization (11, 12).

The literature has analyzed the efficacy and efficiency of telemedicine applied to the oncology field with assessments through systematic reviews, meta-analyses and clinical trials. Specifically, the aim of telemedicine in oncology is to reduce the access disparity and patients care (13). O’Gorman et al. within his own study shows that the use of telemedicine was higher in rural areas of northern Ontario in Canada compared to other parts of the province suggesting that telemedicine is utilized to improve access to medical care services, especially in sparsely populated and least served regions (14).

Patrick D. Hoek et al. (15) through a clinical trial, highlighted the most problematic aspects in the application of teleconsultation, showing how it is necessary to seek ways of optimizing multidisciplinary care done through teleconsultation, making the times and frequency of teleconsultations more suitable for patients with advanced cancer, underlining the need of doing research in this area for improving of the most critical aspects of telemedicine (15).

In Italy, the application of telemedicine in the oncology field has not yet found widespread use despite its great potential. For example the Ministry of Health released in 2014 the national guidelines on telemedicine define the roles and tasks of telemedicine (16).

A recent published document (AIOM, CIPOMO) for COVID-19 emergency indicates the need to postpone the follow-up outpatient activities for disease-free patients (e.g., follow-up to 6- 12 months), providing a telephone triage and/or telematic patient rescheduling based on clinical severity (17). This indication opens the opportunity to apply telemedicine methodologies in emergency situations such as the current one but also in the post-emergency phase.

Advanced computer technology widely available at medical institutions allows a reduction of required human resources and makes the routine collection of data feasible in busy clinical practices (18–21). In particular, computer technology has been extensively applied for immediate record and presentation of PRO results to clinicians, utilizing useful tools for data collection and presentation, already validated and easily transferable to other fields of clinical activities (22–24). This is applicable for research purposes, for symptom screening and patient monitoring thereby contributing to hospital quality assurance.

### COVID Infection and Cancer: Individualized Management

According to the latest data in the literature concerning the characteristics of COVID-19 infection, we can assume that age and comorbidities strongly influence prognosis, and mainly, the possibility to develop a severe disease with respiratory failure and ARDS (1, 25). In particular, some comorbidities, such as chronic obstructive pulmonary disease and cardiovascular diseases place the patients at higher risk of developing a severe form of COVID-19 and ultimately death.

On the other hand, due to the low number of data available, little is acknowledged about the impact of COVID-19 infection in patients affected by cancer. Only few retrospective studies have faced this issue and their results were in favor of a heavy impact of cancer on prognosis. So, it is presumable that COVID-19 tends to have a poorer outcome in cancer patients (26, 27).

Solid tumors are genetically characterized by a strong immune-suppression which at first involves tumor microenvironment, then in a more advanced phase the whole organism. This immune-suppressive status is strongly due to the over production, mainly by the tumor cells, of cytokines such as IL-6, TNF-alpha and TGF-beta (28). Moreover, patients affected by solid tumors often suffer from blood coagulation disorders because of chemotherapy, surgery, hormone therapy, biological agents (e.g., anti-VEGFR drugs), and bedding. In addition, cancer cells are capable of producing the “cancer pro-coagulant” which is a cysteine proteinase able to induce a thrombotic diathesis, mainly in very advanced tumors (29).

Finally, most patients affected by cancer, in particular those suffering from lung cancer, head and neck carcinomas, urologic cancers, and gastrointestinal cancers are elderly patients (>70 years old) and often present a number of comorbidities at diagnosis. Moreover, a fair number of such patients, especially those having lung cancer and head/neck carcinomas, are heavy smokers. Both age and smoker status seem to poorly impact prognosis of patients affected by COVID-19 (30).

### Cancer Patient Management

COVIDd-19 (from now on indicated interchangeably as COVID or COVID-19) is determining a complete reorganization of hospitals with two new different paths for COVID and non-COVID. Many hospital units have been converted to COVID units with the risk that non-COVID patients may not receive adequate therapy for their disease.

The fragility of cancer patients is well known, with many cases affecting aged patients or those with several comorbidities that frequently results in a loss of independency and functionality. Based on these patients characteristics and the data coming from Protezione Civile indicating that around 20% of cancer deaths from COVID infection occurs in patients with previous cancer or active of cancer (1), a number of actions in the hospitals have been set up to maintain cancer patients out of hospitals when there is no urgent need. It has been recommended that, when possible, the follow up visits for cancer patients treated with oral anticancer drugs are performed telematically. Furthermore, many patients refuse hospital visits, even if necessary, because of fear of contagion: in some Italian regions the first non-urgent visits have been postponed with the consequent delay in diagnosis which will affect disease prognosis.

The practical experience of oncologic units could offer assistance in the way of telemedicine that should be organized with appropriate tools in order to plan for personnel, including patient count and a clear definition of patients who may benefit from this approach. Telemedicine may also be useful to increase or preserve cancer screening programs.

It is desirable that this type of experience could produce telematic clinical strategies that will allow, in the Covid-19 post-emergency, to continue caring for cancer patients in an appropriate setting where a number of hospital visits are substituted with visits at small local health centers and general practitioners’ office and taking advantage of well-defined telemedicine path which will be developed in this phase.

## Conclusion

The main conclusion of the above considerations is that patients suffering from solid tumors tend to develop a severe form of COVID-19 infection, which can often lead to death, due to the cancer “*per se*,” and the comorbidities linked to cancer. Thus, the management of patients affected by cancer during COVID-19 pandemic is particularly challenging.

The first precaution to take is to reduce the risk of contagion in these patients. The general policy of Cancer Centres (CC) in Italy has been to remain COVID-19 free to ensure sufficient clinical and intensive-care capacity for critical cancer surgeries or management of side effects from systemic anticancer treatment. This goal could be achieved by immediately transferring the new infected cases from CC to specialized hospitals for the treatment of SARS-CoV-2 infections (Covid-Centers In this scenario, we can hypothesize to act in a similar way.

Importantly, the follow-up of outpatients with telemedicine should be strongly individualized and based on disease severity and initial treatment priorities. The possibility of obtaining diagnostic test results via e-mail, as well as images related to radiological tests cannot be excluded. The contribution of the general practitioner in visiting patients with suspicious symptoms at home should also be considered.

In this pandemic situation a multidisciplinary approach for the management of cancer patients should be improved where possible in order to give the most priority to patients, better equilibrating oncologic and COVID-19 needs.

The management of patients considered infected depends on their symptoms. In asymptomatic patients, it is reasonable to propose quarantine at home and careful telephone monitoring of symptoms. Patients with mild symptoms, such as slight-fever, cough and mild asthenia, should be early hospitalized in CC facilities, since they can deteriorate rapidly. Telemedicine would also allow for prompt treatment of COVID-19 patients with cancer right in their homes with prophylactic heparin treatment and other drugs employed in hospital settings during the early phase of the disease.

## Data Availability Statement

The original contributions presented in the study are included in the article/supplementary material, further inquiries can be directed to the corresponding author.

## Author Contributions

AC and SP: conceptualization and methodology. CM, FP, MG SB, and LA: formal analysis. AC, CM, FP, AA, LA, EC, MC, MD, and SP: investigation. CM, MG, FP, SB, and LA: resources. AC, MC, and SP: supervision. AC, CM, FP, MG, LA, MC, and SP: writing – original draft. AC, MC, LA, and SP: writing – review and editing. All authors have read and agreed this manuscript.

## Conflict of Interest

The authors declare that the research was conducted in the absence of any commercial or financial relationships that could be construed as a potential conflict of interest.
